# Cervical cancer screening: Safety, acceptability, and feasibility of a single-visit approach in Bulawayo, Zimbabwe

**DOI:** 10.4102/phcfm.v7i1.742

**Published:** 2015-05-05

**Authors:** Muriel S. Fallala, Robert Mash

**Affiliations:** 1 Division of Family Medicine and Primary Care, Stellenbosch University, South Africa

## Abstract

**Background:**

Cervical cancer is the commonest cancer amongst African women, and yet preventative services are often inadequate.

**Aim:**

The purpose of the study was to assess the safety, acceptability and feasibility of visual inspection with acetic acid and cervicography (VIAC) followed by cryotherapy or a loop electrical excision procedure (LEEP) at a single visit for prevention of cancer of the cervix.

**Setting:**

The United Bulawayo Hospital, Zimbabwe.

**Methods:**

The study was descriptive, using retrospective data extracted from electronic medical records of women attending the VIAC clinic. Over 24 months 4641 women visited the clinic and were screened for cervical cancer using VIAC. Cryotherapy or LEEP was offered immediately to those that screened positive. Treated women were followed up at three months and one year.

**Results:**

The rate of positive results on VIAC testing was 10.8%. Of those who were eligible, 17.0% received immediate cryotherapy, 44.1% received immediate LEEP, 1.9% delayed treatment, and 37.0% were referred to a gynaecologist. No major complications were recorded after cryotherapy or LEEP. Amongst those treated 99.5% expressed satisfaction with their experience. Only 3.2% of those treated at the clinic had a positive result on VIAC one year later. The service was shown to be feasible to sustain over time with the necessary consumables. There were no service-related treatment postponements and the clinic staff and facility were able to meet the demand for the service.

**Conclusion:**

A single-visit approach using VIAC, followed by cryotherapy or LEEP, proved to be safe, acceptable and feasible in an urban African setting in Bulawayo, Zimbabwe. Outcomes a year later suggested that treatment had been effective.

## Abstrait

Dépistage du cancer du col de l’utérus: Innocuité, acceptabilité et faisabilité de l’approche à visite unique à Bulawayo, Zimbabwe.

**Contexte:**

Le cancer du col de l’utérus est le plus commun parmi les femmes africaines, mais cependant les services de prévention sont souvent inadéquats.

**Objectif:**

Le but de l’étude était d’évaluer l’innocuité, l’acceptabilité et la faisabilité de l’inspection visuelle avec de l’acide acétique et une cervico-graphie (VIAC) suivis d’une cryothérapie ou de la technique d’excision électro-chirurgicale à l’anse (LEEP) en une seule visite pour la prévention du cancer du col de l’utérus.

**Cadre:**

L’hôpital United Bulawayo Hospital, Zimbabwe.

**Méthodes:**

L’étude descriptive a utilisé des données rétrospectives provenant des dossiers médicaux électroniques des femmes qui fréquentent les cliniques de VIAC. Sur une période de 24 mois, 4641 femmes ont fréquenté la clinique et ont été contrôlées pour le cancer du col de l’utérus au moyen du VIAC. Celles dont le résultat était positif ont reçu immédiatement un traitement de Cryothérapie ou de LEEP. Les femmes traitées ont eu un suivi à trois mois et un an.

**Résultats:**

Le taux de résultats positifs avec les tests de VIAC étaient de 10.8%. Parmi les personnes admissibles, 17.0% ont été mises immédiatement sous cryothérapie, 44.1% ont eu immédiatement une LEEP, 1.9% un délai de traitement, et 37.0% ont été envoyées chez un gynécologue. On n’a pas enregistré de complications importantes après la cryothérapie ou la LEEP. Parmi les personnes traitées, 99.5% étaient satisfaites de leur expérience. Seules 3.2% des femmes traitées à la clinique ont eu un résultat positif à la suite de la VIAC un an plus tard. Le service s’est avéré réalisable pour se maintenir au fil du temps avec les produits de consommation nécessaires. Il n’y a pas eu de reports de traitement relatifs au service et le personnel de la clinique et l’équipement ont pu répondre à la demande du service.

**Conclusion:**

L’approche d’une seule visite avec la VIAC, suivie de cryothérapie ou LEEP, s’est avérée être sans danger, acceptable et faisable dans un environnement urbain africain à Bulawayo, Zimbabwe. Les résultats un an plus tard ont montré que le traitement avait été efficace.

## Introduction

Cervical cancer is the commonest cancer amongst African women and globally is exceeded only by breast cancer.^1^ In sub-Saharan Africa cancer of the cervix is thought to account for 22% of all cancers in women, although accurate data are difficult to obtain in most countries.^2^ This cancer claims approximately 270 000 lives of women worldwide each year, and nearly 85% of those deaths occur in resource-poor settings.^1^ The reasons for these high rates are because preventive strategies and treatment are not carried out well in developing countries. In resource-limited countries, like Zimbabwe, a shortage of skilled health workers, lack of political will and insufficient funds for women's health activities contribute to these high rates.^3^

Treatment of advanced cancer is extremely difficult in most developing countries, whilst successful treatment of pre-invasive cancer should be technically possible.^4^ Introduction of the Papanicolau smear has successfully decreased the incidence of invasive cancer and mortality in developed countries.^4^ However, even screening is difficult to implement in developing countries because of poverty, poor infrastructure, lack of financial and human resources, and the disempowerment of women.^3^

New methods for screening, such as human papillomavirus testing and visual inspection of the cervix with acetic acid (VIA),which are adaptable to limited-resource countries, have been investigated.^3^

VIA, also known as direct visual inspection, ‘involves examining the cervix with the naked eye, using a bright light source, after the application of 3% – 5% dilute acetic acid using a cotton swab or a spray’.^3^ Acetic acid is a component of ordinary vinegar and therefore widely available. VIA provides health workers with an immediate result in the clinic, whilst cytology requires a laboratory report. This approach therefore saves time, allows treatment to be given immediately, reduces anxiety about future test results, and does not require further investigations.^3^ Primary healthcare is therefore strengthened by improved access to a more comprehensive service.

Cervicography is taking a digital picture of the cervix after VIA, using a fairly ordinary camera mounted with a special lens.^5,6,7^ The photographs are viewed on a television or computer screen and interpreted by trained nurses or doctors.^6,7^ Cervicography magnifies the cervix so that it can be viewed on a large screen. It also provides a permanent record that can be discussed later with colleagues, can be shown to the client, and can be compared with previous pictures. Cervicography has improved the sensitivity and specificity of VIA. The greatest criticism of VIA has always been quality control: VIA with cervicography (VIAC) has resolved this, as there is now a visual record that can be discussed by colleagues and transmitted online.

The goal of treatment of precancerous lesions is to remove the lesion, and this can be accomplished by either ablation (cryotherapy or cautery), loop electrical excision procedure (LEEP), cone biopsy or hysterectomy.^8,9,10^ All of these treatment methods have a good success rate; the choice of treatment depends on the size or extent of the lesion, client acceptability, reproductive needs, cost, availability of equipment and expertise. Since VIAC is based on a single visit ‘see and treat’ approach, only treatment modalities that fit into this approach are discussed further: cryotherapy, cautery, LEEP and cone biopsy.^8,9,10^

**FIGURE 1:**
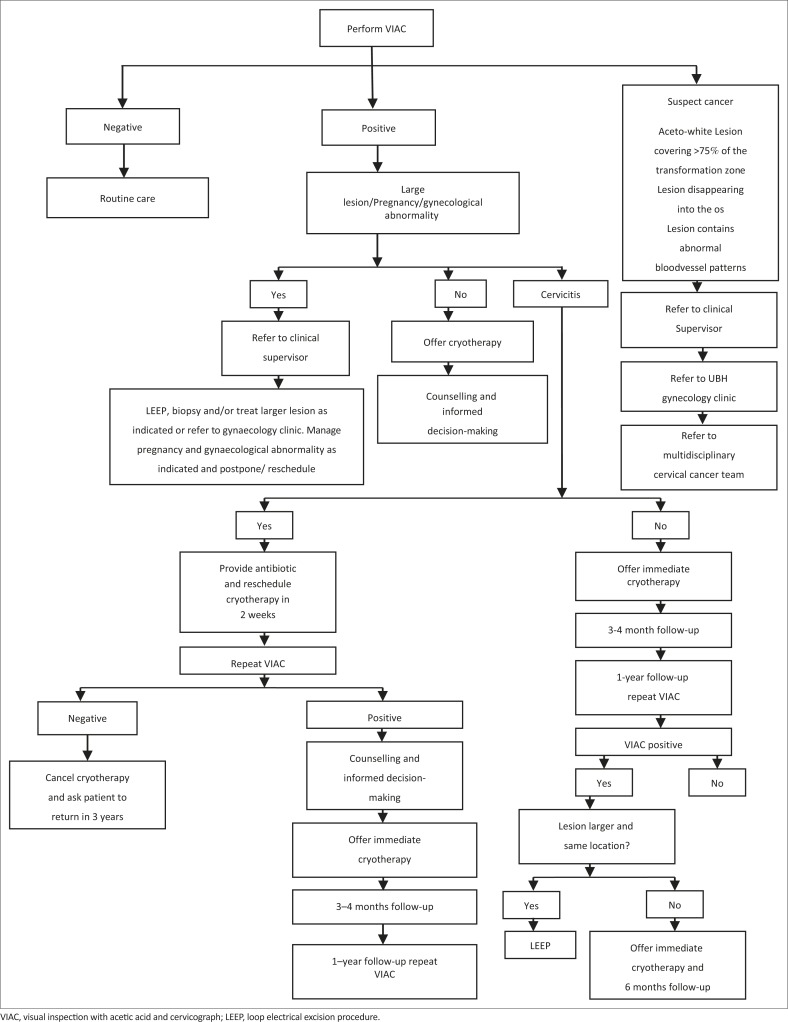
Clinical protocol for Zimbabwe safety, acceptability and feasibility project. VIAC, visual inspection with acetic acid and cervicograph; LEEP, loop electrical excision procedure.

Cryotherapy of the cervix is a method of destroying precancerous cells by cold coagulation using ice-cold gas and no local anesthesia.^8,9^ The main disadvantage is that there is no histological specimen available for evaluation. If an aceto-white lesion is visualized during VIAC, cryotherapy treatment can be performed following set criteria as shown in Figure 1.^11,12,13,14^

LEEP is also called large loop electro-diathermy excision procedure, and is performed under local anesthesia using 2–5 mL lignocaine without adrenaline at 3-, 6-, 9-, and 12 o'clock positions. It entails using a wire with an electrical current passing through it to cut and remove tissue under direct vision.^8^ The wire cuts and simultaneously coagulates. The transformation zone, including the lesion, is therefore removed. This can be used as both a diagnostic or therapeutic procedure.

Criteria for LEEP are as follows:^8^

- Aceto-white lesion covering > 75% of the transformation zone.
- Lesion disappearing into the os.
- Lesion contains abnormal blood vessel patterns.
- Persistent lesion after cryotherapy.
- Failure of agreement between cytology, VIAC and histology.
- The limits of the lesion in the cervix cannot be completely defined by VIAC.

The main value of the study was to investigate whether a single-visit approach to cervical cancer screening is acceptable and feasible to staff and patients, and the treatment options are safe. Similar projects have been undertaken in Ghana,^6^ Zambia^8^ and Thailand.^13^

In Zambia the high proportion of women (67%) that accepted screening for cervical cancer and the high proportion of women completing the referral show that existing HIV prevention interventions provide a springboard for reaching women rapidly with timely, life-saving and easily adaptable cervical cancer prevention and treatment programmes such as ‘screen and treat’.^9^

Both the Ghanaian and the rural Thailand studies assessed the safety, acceptability and feasibility of cryotherapy performed by trained nurses. The knowledge gap to be addressed was to find out if this approach is acceptable and safe to those accessing it in the Zimbabwean context, and also to evaluate if it is feasible to offer such a service effectively in the context of the poorly resourced health system. There will be a need to look at certain aspects of the screening process, such as follow-up care of patients with abnormal results, sustainability of the programme and whether the programme meets the screening standards set by the World Health Organization. The findings of the study will be discussed with the Ministry of Health and relevant policy makers, and will add knowledge required to develop effective health policies in Zimbabwe on an important aspect of women's health.

### Aim and objectives

The aim of the study was to evaluate VIAC screening and immediate intervention (cryotherapy or LEEP) for the prevention of cervical cancer amongst women attending the United Bulawayo Hospital in Zimbabwe. The objectives of the study were:

- To evaluate the safety of this approach- the proportion experiencing severe bleeding, shock or requiring hospitalisation during treatment, proportion with post-treatment complications, and proportion returning for an additional visit due to problems.
- To evaluate patient satisfaction with and adherence to this approach – the proportion that consented at their single visit, proportion of treatment postponed in order to consult with family members, proportion attending follow-up appointments, proportion whose partners abstained from sex for four weeks after treatment by successfully adhering to home care instructions, and proportion satisfied with the screening and treatment service.
- To evaluate the technical feasibility of this approach – treatment performance rate in those who were eligible, and proportion with treatment postponed due to break down of equipment.
- To evaluate the clinical outcome – VIAC-positive rate at one year post-treatment at the clinic.

## Research methods and design

### Study design

This was a descriptive cross-sectional study using retrospective data from the medical records of women attending the VIAC clinic at United Bulawayo Hospital in the period 2010–2012.

### Setting

The study was carried out in a VIAC clinic located at United Bulawayo Hospital. The VIAC clinic was incorporated into an already existing Family Planning clinic. The clinic has been functional since June 2009 and has four rooms. One room was for data capturing, counselling for VIAC, contraception and HIV services, and testing for HIV infection. The second room was a procedure room for VIAC and cryotherapy. The third room was a small theatre for LEEP, and the fourth room was a toilet. The clinic employed four midwives trained in counselling, VIAC, cryotherapy and family planning. Registrars, gynaecologists and junior doctors trained in VIAC, cryotherapy and LEEP also worked in this clinic.

The duration of the training course was 14–21 days. All nurses were expected to master VIAC and cryotherapy. Doctors were expected to master skills to perform VIAC, cryotherapy, LEEP, cautery, cone and punch biopsy, provided they worked in institutions where complications of those procedures can be dealt with adequately. The participants were trained by two gynaecologists and two senior registrars. Training was competency-based and involved observation and feedback on an ongoing basis.

The guideline followed by the clinic was that those who were HIV negative were screened for cervical cancer every 3 years whilst those who were HIV positive were screened once a year.^3^ The recommended optimal age for screening was defined as 30–39 years of age, expanding to 18–65 years as resources permitted. Those women who received cryotherapy or LEEP were reviewed 4 weeks post-treatment and then a year later. Figure 1 shows the flow of patients in the VIAC clinic. Patients with cancer of the cervix were referred to the gynaecology clinic at the local referral hospital.

### Study population

The study population included all women (4641) who were screened during the period 2010–2012. No pregnant women are screened at the clinic.

### Data collection

Data were collected by the researcher and three research assistants (another doctor and two clerks) from patients’ electronic medical records and captured in an Excel spreadsheet. Data were collected to calculate the following variables:

- Proportion of VIAC-positive women.
- Proportion of women who were offered and accepted immediate post-VIAC treatment.
- Proportion of women who postponed treatment versus number of women who accepted it immediately.
- Proportion of women who developed complications during treatment.
- Proportion of women who developed complications after treatment (up to 6 weeks).
- Proportion of women satisfied with the service.
- Proportion of women supported by their spouses by abstaining from sex for 4 weeks following treatment.
- Proportion of women found with invasive cervical cancer on first VIAC examination.
- Proportion of women returning for review after 12 months post-treatment.
- Proportion of women found positive on VIAC upon follow-up after treatment.

On arrival at the clinic patients were counselled for VIAC and HIV testing. At the end of the VIAC procedure patients were presented with their results, and at the same time they were asked if they were satisfied with the decision they had made to be tested. This was recorded in their notes and used to calculate the proportion of women who were satisfied with the service.

### Data analysis

Data were analysed using Excel and Epi-Info software to generate tables and calculate frequencies, proportions, means and standard deviations (SD) with 95% confidence intervals.

### Ethical considerations

Permission was obtained from the Health Authority of United Bulawayo Hospital to conduct the research, and ethical approval was obtained from the Health Research Ethics Committee of Stellenbosch University (Ref. No. HREC S13/02/036). Since there was no direct contact with the women and only the electronic medical records were used, there was a waiver of informed consent.

## Results

Two nurses screened 4641 women using the protocol shown in Figure 1 and 501 screened VIAC positive. All ages in the targeted range were represented; the mean age was 39 (SD 11.3) years, as shown in Table 1. Most patients (61.2%) attended the clinic with one or more of the following complaints: lower abdominal pain, vaginal discharge, vaginal bleeding, backache, dyspareunia or vaginal itchiness. Only 17.4% of women reported ever having had a Pap smear before. More than half (52.5%) of those screened tested HIV positive; some of them came to the clinic with their results whilst others were screened at the clinic on the VIAC day. Ten per cent did not know their HIV status, meaning that they had refused testing. The mean age of sexual debut was 18 (SD 3.7) years, and contraception was used by 61.9% of these women.

**TABLE 1:** Programmatic factors and cervical cancer risk factors in relation to VIAC positive results (*N* = 501).

Variable	Frequency	Percentage
**Marital status**		
Single	69	13.8
Married	315	62.9
Widowed	89	17.8
Divorced	28	5.6
**Previous Pap smear done**		
Yes	87	17.4
No	414	82.6
**HIV Status**		
Positive	263	52.5
Negative	188	37.5
Unknown	50	10.0
**Initial complaints**		
Vaginal bleeding	47	9.4
Vaginal discharge	41	8.2
Lower abdominal pain (LAP)	107	21.4
LAP and lower back pain	52	10.4
LAP and watery discharge	1	0.2
Lower back pain	6	1.2
Heavy menstruation	1	0.2
Post-coital bleeding	1	0.2
Watery vaginal discharge	6	1.2
LAP and vaginal bleeding	3	0.6
Dyspareunia	1	0.2
**Use of contraceptives**		
Condoms	87	17.4
Oral contraceptives	102	20.4
Levonorgestrel implant	27	5.4
Tubal ligation	10	2.0
Depot progesterone injection	44	8.8
Intra-uterine contraceptive device	15	3.0

LAP, Lower abdominal pain.

**FIGURE 2:**
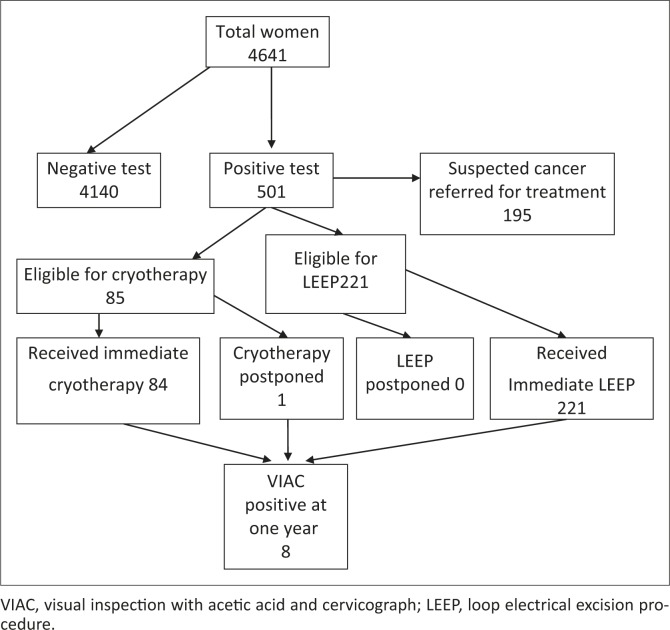
Patient flow in visual inspection with acetic acid and cervicograph clinic. VIAC, visual inspection with acetic acid and cervicograph; LEEP, loop electrical excision procedure.

Overall 10.8% of the women were positive on VIAC and 195/501 (38.9%) of those testing positive had suspected cancer or other problems requiring referral to a gynaecologist (see Figure 2).The large number of referrals to the gynaecologist could have been due to the fact that this was a new clinic and many symptomatic women were able to access the service for the first time. As shown in Table 2, 85/501(17.0%) of VIAC-positive women were eligible for immediate treatment with cryotherapy, and of these 98.1% received cryotherapy during the project period. On the other hand, 221/501(44.1%) of VIAC-positive women were eligible for immediate treatment with LEEP, and of these 100% received LEEP during the project period.

**TABLE 2:** Selected clinical and programmatic outcomes.

Variable	*N*	%
**Screening (*N* = 4641)**		
VIA test positive	501	10.8
Satisfied with their decision to be tested	4641	100.0
**Cryotherapy (*N* = 85)**		
Accepted immediate offer of cryotherapy	84	98.8
Total cryotherapy performed amongst those eligible	84	98.8
Treatments postponed due to staff- or facility-related issues	0	0.0
Delayed treatment due to patient	1	1.9
Clinic visit for perceived problem	0	0.0
Major complications (bleeding, shock, hospitalisation)	0	0.0
Satisfied with their decision to be treated	84	97.7
Complied with post-cryotherapy instructions	82	97.7
Attended follow-up after one year	85	100.0
Tested positive at one year	1	1.2
**LEEP (*N* = 221)**		
Accepted immediate offer of LEEP	221	100.0
Total LEEP performed amongst those eligible	221	100.0
Treatments postponed due to staff- or facility-related issues	0	0.0
Delayed treatment due to patient	0	0.0
Clinic visit for perceived problem	0	0.0
Major complications (bleeding, shock, hospitalisation)	1	0.5
Satisfied with their decision to be treated	218	98.6
Complied with post-LEEP instructions	218	98.6
Attended follow-up after one year	213	96.4
Tested positive at one year	7/213	3.2

VIAC, visual inspection with acetic acid and cervicograph; LEEP, loop electrical excision procedure.

All of the treated patients were followed up immediately after treatment, and only one of the women who was treated experienced complications. With regard to their husbands or partners, 97.7% of women who had received cryotherapy and 98.6% of those receiving LEEP were able to negotiate abstaining from sex in their relationships for 4 weeks. After treatment 97.7% were satisfied with their decision to be treated with cryotherapy whilst 98.6% were satisfied about their decision to be treated with LEEP.

One year later 96.4% of the women who had received LEEP came back for review and 100% of the women who had received cryotherapy. At one year after treatment the VIAC-positive rate was low, at 1.2% after cryotherapy and 3.2% after LEEP.

## Discussion

The VIAC test was positive in 10.8% of all the women screened, which may be higher than expected due to the fact that this was a new service and most women would not previously have had the opportunity to be screened. Of those who were eligible, 17.0% received immediate cryotherapy, 44.1% immediate LEEP, 1.9% delayed treatment, and 37.0% were referred to a gynaecologist. No major complications were recorded after cryotherapy or LEEP. Amongst those who were treated, 99.5% expressed satisfaction with their experience. Only 2.6% of those who were treated at the clinic were VIAC-positive one year later.

This study therefore shows that VIAC followed by treatment with either cryotherapy or LEEP is safe, acceptable, feasible and effective in a poorly resourced setting such as Bulawayo, Zimbabwe. The study supports the viewpoint that this approach should be considered as an alternative to prevention programmes based on cytology in areas where technical, infrastructural, and financial barriers exist.^5,6,7^ The safety and effectiveness of VIAC, cryotherapy and LEEP performed by nurses and junior doctors in this study was also seen in studies from Zambia, Ghana, India andThailand.^6,8,13^

The need for such task shifting to nurses and junior doctors in our setting is important because of the lack of human resources for health. As part of routine quality assurance, aceto-white lesions one year post- cryotherapy or LEEP is an indication for retreatment or referral. Positive VIAC test rates amongst the women who returned after cryotherapy and LEEP were 1.2% and 3.2% respectively. VIAC is known to have a high negative predictive value in primary care settings (at least 96%), and therefore these low rates on follow-up suggest that cancers were successfully treated.^15,16,17^

The approach evaluated in this study was also acceptable to women, and there was good adherence to the programmatic requirements for follow-up and abstinence. Adherence to post-procedure instructions and follow-up was higher than in a similar study in Ghana, which might be due to the emphasis on counselling in this clinic in Bulawayo.^6^ A further study should look into the factors which resulted in a good follow-up rate, as this is often cited as a barrier to effective screening programmes in developing countries.^17^

Feasibility of sustaining and providing the service was also good, and even in Zimbabwe it was possible to obtain the gas supply for cryotherapy, as well as the consumables necessary for VIAC and LEEP in this urban setting. There were no service-related treatment postponements and the clinic staff and facility were able to meet the demand for the service. VIAC with immediate cryotherapy or LEEP has also been shown to be cost-effective in other settings.^16^

If screening was able to reach 70% of the target population over a 5–10-year period, then mortality could be reduced by 25%.^6^ It is not possible to calculate an accurate population coverage rate from the data in this study. Future studies should look at how to achieve such population coverage and measure the impact on morbidity and mortality, as well as investigate the cost, sustainability and quality assurance of such a service.^5,6,7^

## Conclusion

The results indicate that a single-visit approach based on VIAC, cryotherapy and LEEP performed by trained nurses and junior doctors respectively is safe and feasible in this low-resourced urban setting in Zimbabwe. This approach was also acceptable to the women and there was good adherence to the programmatic requirements for follow-up and abstinence. The treatment outcomes one year later were favourable, with an overall VIAC-positive test rate of only 2.6%. Policymakers in this and similar low- resource settings should consider prevention programmes for cervical cancer based on this approach, rather than adopting models from more developed countries.
